# Florid cystitis glandularis (intestinal type) with mucus extravasation: Two case reports and literature review

**DOI:** 10.3389/fsurg.2023.1048119

**Published:** 2023-02-24

**Authors:** Tao Zhang, Si-Fan Yin, Wen-Bo Feng, Chang-Xing Ke

**Affiliations:** Department of Urology, The Second Affiliated Hospital of Kunming Medical University, Kunming, China

**Keywords:** cystitis glandularis, intestinal type, diagnosis, treatment, clinical features

## Abstract

**Background:**

Cystitis glandularis is a common bladder epithelial lesion characterized by hyperplasia and metaplasia of the bladder mucosa epithelium. The pathogenesis of cystitis glandularis of the intestinal type is unknown and less common. When cystitis glandularis (intestinal type) is extremely severely differentiated, it is called florid cystitis glandularis (the occurrence is extremely rare).

**Case summary:**

Both patients were middle-aged men. In patient 1, the lesion was also seen in the posterior wall and was diagnosed more than 1 year ago as cystitis glandularis with urethral stricture. Patient 2 was examined for symptoms such as hematuria and was found to have an occupied bladder; both were treated surgically, and the postoperative pathology was diagnosed as florid cystitis glandularis (intestinal type), with mucus extravasation.

**Conclusion:**

The pathogenesis of cystitis glandularis (intestinal type) is unknown and less common. When cystitis glandularis of the intestinal type is extremely severely differentiated, we call it florid cystitis glandularis. It is more common in the bladder neck and trigone. The clinical manifestations are mainly symptoms of bladder irritation, or hematuria as the main complaint, which rarely leads to hydronephrosis. Imaging is nonspecific and the diagnosis depends on pathology. Surgical excision of the lesion is possible. Due to the malignant potential of cystitis glandularis of intestinal type, postoperative follow-up is required.

## Introduction

Cystitis glandularis (CG) is a proliferative disease of the bladder mucosa characterized by transitional cell glands. CG was first described by Morgagni et al. in 1761 (1). There are two types based on microscopic features: (1) common type and (2) intestinal type. The pathogenesis of cystitis glandularis (intestinal type) is unknown and less common. When cystitis glandularis (intestinal type) is extremely severely differentiated, it is called florid cystitis glandularis (the occurrence is extremely rare). We retrospectively analyzed the clinical and pathological data of two patients in our hospital to analyze the disease, with the aim of further deepening the understanding and thus improving the diagnosis and treatment of the disease.

### Case introduction

#### Case 1

**Figure 1:**
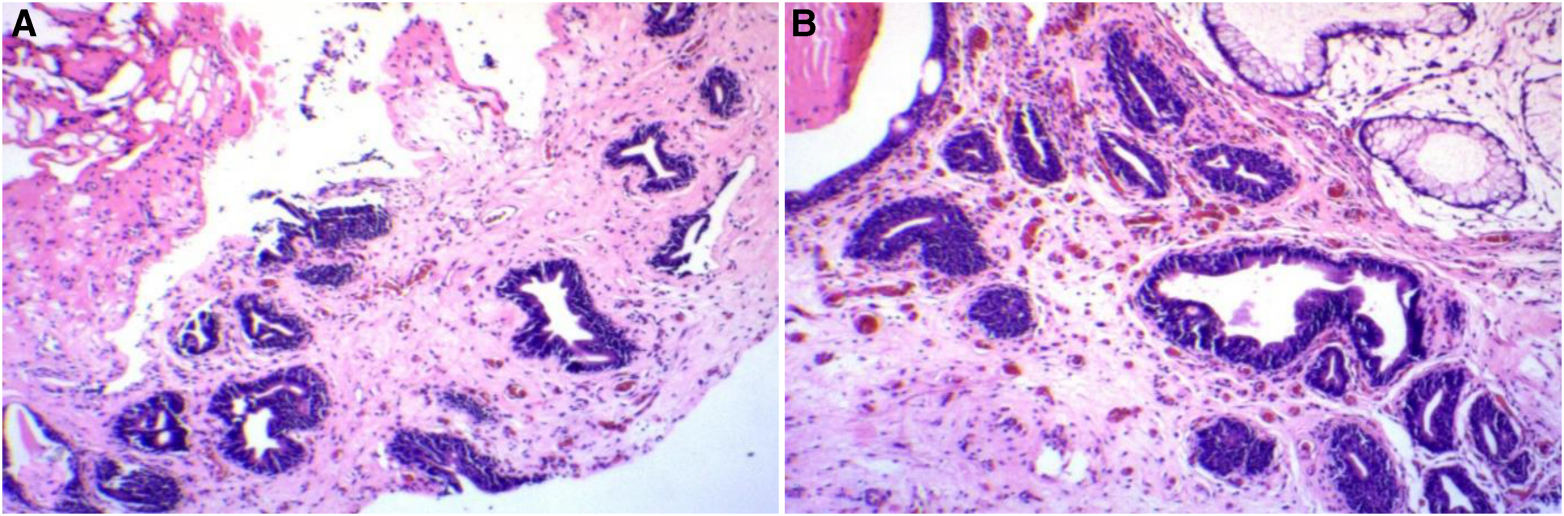
Patient 1’s pathology and immunohistochemistry. (**A**) Hematoxylin–eosin staining. (**B**) Hematoxylin–eosin staining.

A 44-year-old man presented with a diagnosis of cystitis glandularis 1.5 years ago. The patient underwent holmium laser lithotripsy and urethral dilatation for urethral stricture and bladder stone interruption at an outside hospital. Since a new organism was found intraoperatively, a tissue biopsy was performed. The pathological diagnosis was cystitis glandularis. The patient was referred to our hospital for further treatment. The patient denied a history of a long-term indwelling urinary catheter, long-term exposure to chemical substances, and denied ever smoking. There was a history of hepatitis B and, therefore, abnormal liver function. Ultrasound of the bladder showed roughness of the bladder wall. The bladder wall was thickened, and a contrast-enhanced CT scan showed enhancement. The lesion was excised, and the urethral stricture was dilated. The lesion was located in the bladder triangle and posterior wall. The lesion was lamellar, and the urine spraying at the bilateral ureteral orifices showed no abnormality. Postoperatively, the soft tissue was grayish white. The differentiation from von Brunn's nest was it was more dense and irregular, with different morphology, distinct atypical hyperplasia, significantly enlarged lumen, and mucus lakes in the interstitium (as shown in Figure 1). The pathological diagnosis was florid cystitis glandularis (intestinal type) with mucus extravasation. Immunohistochemistry showed the following: CK7 (+), CDX-2 (+), Ki-67 (about 3%), and GATA3 (+). We followed up with this patient. To date, no recurrence has been observed.

#### Case 2

**Figure 2:**
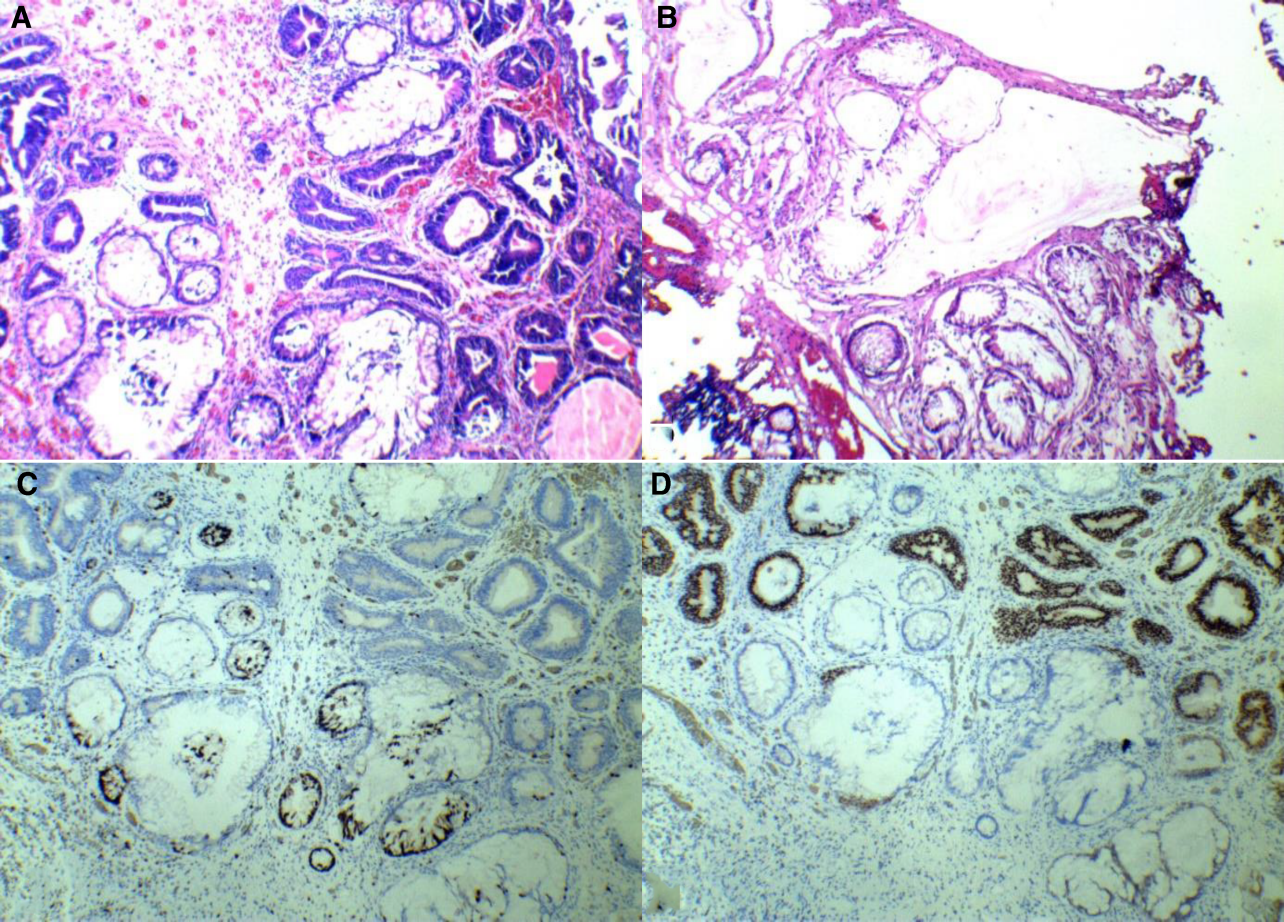
Patient 2’s pathology and immunohistochemistry. (**A**) Hematoxylin–eosin staining. (**B**) Hematoxylin–eosin staining. (**C**) CDX-2(+). (**D**) GATA-3(+).

A 46-year-old man presented with “lumbar pain, lower abdominal pain with hematuria for more than 1 month.” It was a painless hematuria throughout, containing small amounts of blood clots and putrefied tissue. Ultrasound and CT showed a left ureteral stone, left kidney stone, and bladder occupancy (outside hospital). He had a history of hypertension. There was no history of a long-term indwelling urinary catheter, no history of long-term toxic exposure, and the rest was not exceptional. Urine routine showed erythrocytes 7,468/μL. Urine microscopy showed erythrocytes ++++ (>100)/HP. CT scan showed thickening of the right bladder wall. Contrast-enhanced CT scans showed enhancement. After adequate preoperative preparation, resection of the bladder mass was performed. Intraoperatively, the lesion was seen to be located in the bladder triangle and at the opening of the right ureter. The basal width of the lesion was approximately 3 cm × 3 cm, and no blood spray was seen at the left ureteral opening. Due to the wide resection of the right ureteral opening, one F6 ureteral stent tube was placed in the right ureter. The postoperative naked eye view is grayish-gray-brown tissue. The differentiation from von Brunn's nest was it was more dense and irregular, with different morphology, distinct atypical hyperplasia, significantly enlarged lumen, and mucus lakes in the interstitium. The pathological diagnosis was florid cystitis glandularis (intestinal type) with mucus extravasation. Immunohistochemistry showed the following: GATA3 (+), CK7 (+), CDX-2 (+), and Ki-67 (10%) (as shown in Figure 2). We followed up with this patient. To date, no recurrence has been observed.

## Related literature learning

Cystitis glandularis is a common bladder epithelial lesion characterized by hyperplasia and metaplasia of the bladder mucosa epithelium. Cystitis glandularis was first reported in 1761 (2). There are two types based on microscopic features: (1) common type and (2) intestinal type. Cystitis glandularis of the intestinal type was reported in 1942 (3). The etiology and mechanisms of cystitis glandularis are not fully understood, with lower urinary tract infections, obstruction, and other chronic diseases as risk factors.

The pathogenesis of cystitis glandularis has been controversial, but there are three main accepted mechanisms:(1) the embryonic origin theory: during bladder development, ectopic embryonic remnants due to congenital anatomical or abnormal factors can form glands, leading to cystitis glandularis; (2) the theory of epithelial transformation: under long-term pathogenic factors, it can lead to the transformation of the uroepithelium into the glandular epithelium and intestinal epithelial metaplasia; (3) epithelial cells undergo degeneration to an already differentiated previous stage (4). The most widely accepted theory is epithelial transformation. It is believed that the uroepithelium can form von Brunn's nests under certain circumstances and eventually form cystitis glandularis (4). The pathogenesis of cystitis glandularis of the intestinal type is unknown and less common.

When cystitis glandularis (intestinal type) is extremely severely differentiated, it is called florid cystitis glandularis (the occurrence is extremely rare). It is more common in women than men, and cystitis glandularis of the intestinal type is more common in the bladder neck, and trigone. The clinical manifestations are mainly symptoms of bladder irritation or hematuria as the main complaint, which rarely leads to hydronephrosis (5, 6). All cases in this group were diagnosed with florid cystitis glandularis (intestinal type) with mucus extravasation, and we can see that the differentiation from von Brunn's nest is it is more dense and irregular, with different morphology, distinct atypical hyperplasia, significantly enlarged lumen, and mucus lakes in the interstitium. Both patients were middle-aged males with lesions mainly located in the trigone region of the bladder. In patient 1, the lesion was seen in the posterior wall. He was diagnosed with cystitis glandularis with urethral stricture more than a year ago, so this may be a risk factor for its occurrence. Patient 2 had significant ureteral orifice involvement, but the lesion at the ureteral orifice was diagnosed as cystitis glandularis. He had no history of urinary stone disease. Previous reports are generally concordant with our findings. Macroscopically, it appears more often as a polyp or papillary lesion. The common type is lined by columnar or cuboidal cells that do not contain mucin. The lesion is clearly demarcated from normal tissue (3). Cystitis glandularis of the intestinal type is a structure containing intestinal epithelial chemotaxis glands within the lamina propria. It consists of a large number of mucus-secreting cup-shaped cells, which can also coexist with the common type. The intestinal type is predominant, and mucus may extravasate into the interstitium, forming pools of mucus. However, the extravasation of mucus is less common (7); it can also present with inflammation and edema. In histology, mucin is not easily distinguishable from colonic mucin (6).

The formation of von Brunn's nests was visible on microscopic examination in all cases in this group, along with extravasation of mucus and extremely severe differentiation. This is consistent with previous literature reports. Ultrasound, CT, and MRI can all detect lesion bladder wall thickening. It can be found with a nodular or diffuse appearance (8). Imaging studies and cystoscopy cannot distinguish it from malignancy (6, 8). In this group, ultrasound showed roughness of the bladder wall. A plain CT scan + enhancement showed bladder wall thickening with enhancement. The diagnosis of cystitis glandularis of the intestinal type depends on cystoscopy and a pathological analysis of samples. It is easily misdiagnosed as adenocarcinoma (6). It needs to be differentiated from the following diseases: (1) adenocarcinoma of the bladder: the microscopic presentation was mostly arranged in irregular adeno-tubular-like structures with relatively obvious cellular heterogeneity. Under the microscope, glands are arranged neatly. The glands do not invade the detrusor muscle and lack atypical epithelial cells around the mucin. When adenocarcinoma of the bladder is associated with mucus extravasation, tumor cells may be found in or around the mucus pool (6). (2) Bladder endometriosis lesions: it is common in women of childbearing age and rarely occurs in bladder. The main symptoms are abnormal uterine bleeding, hematuria, and suprapubic pain. The bladder triangle and both sides of the bladder wall are frequent occurrence sites. It may be present alone or in combination with other glands (9). Surgical excision and pathologic analysis of the lesion are essential for definitive diagnosis. Cystitis glandularis can be treated by drugs and surgery. Drugs include antibiotics, nonsteroidal anti-inflammatory drugs, and cyclooxygenase-2 inhibitors. The lesion can be excised. A partial or total cystectomy is required for the treatment of refractory cystitis glandularis (10, 11). Florid cystitis glandularis has malignant potential. It is associated with an unknown risk of developing bladder cancer.

However, cystitis glandularis is not a high risk factor for adenocarcinoma and uroepithelial carcinoma. The natural history of this disease is unknown. Both patients were treated surgically and had a good postoperative prognosis.

The pathogenesis of cystitis glandularis (intestinal type) is unknown and less common. When cystitis glandularis (intestinal type) is extremely severely differentiated, it is called florid cystitis glandularis (the occurrence is extremely rare). It is more common in women than in men, and cystitis glandularis of the intestinal type is more common in the bladder neck and trigone. The main clinical manifestations often include hematuria and bladder irritation. However, it rarely leads to hydronephrosis. The presentation is often nonspecific and pathology is the best diagnostic modality. Surgical excision of the lesion is possible. The differentiation from von Brunn's nest is it is more dense and irregular, with different morphology, distinct atypical hyperplasia, significantly enlarged lumen, and mucus lakes in the interstitium. Due to the malignant potential of cystitis glandularis of the intestinal type, postoperative follow-up is required.

## Data Availability

The original contributions presented in the study are included in the article/Supplementary Material, further inquiries can be directed to the corresponding author.
